# Motivating Women to Travel in India: Embodying Safety as an Organizational Purpose

**DOI:** 10.3389/fpsyg.2022.883593

**Published:** 2022-05-13

**Authors:** Raina Chhajer, Vedika Lal, Ankita Tandon

**Affiliations:** ^1^Indian Institute of Management Indore, Indore, India; ^2^VU Amsterdam, Amsterdam, Netherlands; ^3^International Management Institute New Delhi, New Delhi, India

**Keywords:** purpose, safety risk perceptions, travel motivation, women, India

## Abstract

Safety concerns are a key factor that demotivate women from traveling. Tourism organizations are yet to develop approaches to address this comprehensively. Employing the case study design, this study describes how an Indian tourism organization adopted safe women travel as its purpose to reduce women’s safety risk perceptions and motivated them to travel. Nine qualitative interviews were conducted with key stakeholders including co-founders, employees, customers, and vendors. Data were analyzed using thematic analysis resulting in the identification of purpose as a pull factor. Themes of defining, communicating, embodying purpose, and its resulting influence were identified. Through this process, the organization was able to positively impact perceptions of safety, enhance women’s travel motivation, and develop long-term associations with all stakeholders. An actionable framework for implementing purpose was developed that can be used to align tourism organizations’ practices and activities.

## Introduction

Women are increasingly choosing to travel independently, either alone (Seow and Brown, 2018) or in all-female groups (Khoo-Lattimore and Prayag, 2018). They differ from men in their travel preferences, needs (Junek et al., 2006), and motivations (McNamara and Prideaux, 2010). These aspects can help create service offerings specific for women (McNamara and Prideaux, 2010), but they have not been adequately examined (Small et al., 2017). One of the most important issues impacting women’s travel motivations is safety risk (Wilson and Little, 2008; Yang et al., 2017). Women are more vulnerable to crimes especially those that are sexual in nature (Brown and Osman, 2017). This demotivates several women from traveling or constrains their travel choices (Wilson and Little, 2005). All-female tours reduce such risks and are gaining popularity (Khoo-Lattimore and Prayag, 2018). They are pitched as being higher on safety by providing women-specific services. Most travel companies and destination managers focus on cosmetic aspects to customize all-female tour packages, such as women-friendly floors, or women-specific add-ons in accommodations (Yang et al., 2018). These measures, while useful, do not address the core issues of reducing safety risk sustainably. Tourism organizations serious on encouraging more women to travel need to go beyond these cosmetic changes to engage more deeply with the issue and create a safer travel environment for women (Yang et al., 2018).

This study illustrates how Gotravel (pseudo name), a travel organization in Bengaluru, a city in India, endeavored to address this issue seriously by identifying and implementing safe women travel as its purpose. It embodied women safety in its behavioral norms, which reflected in its interactions with customers and motivated women to travel without being conservative in their choices of destinations and experiences. All direct and indirect interactions of prospective women travelers with Gotravel reflected its purpose and *pulled* them to travel with it. Thematically analyzing the data employing push-pull theory of travel motivation (Dann, 1977), we identified organizational purpose as a strong pull factor to motivate women to travel. We present a framework illustrating how Gotravel defined and implemented purpose. Our findings can guide tourism organizations to develop and implement purpose through norms and processes in order to promote safer women travel and enhance their travel motivation.

## Theoretical Background

Individuals’ travel behavior is influenced by their travel motivations, perceived risks, and travel-related constraints (Sönmez and Graefe, 1998; Huang and Hsu, 2009; Chen et al., 2013). For women travelers, perceived safety concerns and socio-cultural constraints are critical factors that often prevent them from traveling (Wilson and Little, 2005; Brown and Osman, 2017; Yang et al., 2017; Khan et al., 2019). The impact of these risk perceptions on travel behavior can be reduced by identifying factors that motivate women to travel (Khan et al., 2019). For example, intrinsic motivators such as feelings of empowerment and independence (Green and Singleton, 2006; McNamara and Prideaux, 2010) encourage women to travel despite the risks involved. In addition, tourism organizations can create extrinsic motivators.

### Motivation to Travel

Several theories have examined travel motivation, of which the push-pull theory (Dann, 1977), one of the most popular approaches to examine travel motivation (Yoon and Uysal, 2005; Khoo-Lattimore et al., 2019), forms the basis of our study. This theory purports that individuals are intrinsically motivated by socio-psychological needs that “push” them and are extrinsically motivated by destination-related factors that “pull” them to travel (Jang et al., 2009; Mohammad and Som, 2010). In one of the earliest studies, Crompton (1979) identified seven push factors (escape, self-exploratory, relaxation, prestige, regression, kinship-enhancement, and social interaction) and two pull factors (novelty and education). Over the years, several factors have been identified that vary based on tourist demographics such as age, marital status, income, education, health status (e.g., Zimmer et al., 1995; Sangpikul, 2008; Hanafiah et al., 2010), nationality (e.g., Jang and Cai, 2002; Hikmah et al., 2013), and destination (e.g., Park et al., 2010; Yousefi and Marzuki, 2015).

Travel experiences vary according to gender (Wilson and Little, 2008). The needs and travel preferences of women differ from men (Junek et al., 2006), as do their tourist experiences (Brown et al., 2020). Thus, their motivations to travel also vary and need examination (Chiang and Jogaratnam, 2006; McNamara and Prideaux, 2010).

### Women Travel Constraints, Motivators, and All-Female Tours

Several factors demotivate women from traveling. They encounter gender-based power differences, gender roles, and behavioral norm expectations while traveling (Brown et al., 2020). They face constraints related to socio-cultural issues and family commitments (Henderson, 1991; Wilson and Little, 2005, 2008). More critically, they confront safety and security risks arising from sexualized male attention (Jordan and Aitchison, 2008; Brown and Osman, 2017). They are also vulnerable to risks involving violent crime, harassment, and theft (Amir et al., 2015; Brown and Osman, 2017; Yang et al., 2017).

All-female tours address several of these constraints and are becoming popular across the world (Gibson et al., 2012; Khoo-Lattimore and Prayag, 2018; Khoo-Lattimore et al., 2019). While providing safety in numbers (Song, 2017), they incorporate several push factors that motivate women to travel. Women are able express themselves freely and perceive a sense of equality (Doran, 2016). They can let go of their gender roles and familial responsibilities (Jennings, 2005), and rediscover their “selves’ (Kasanicky, 2009; Berdychevsky et al., 2013). They escape from routines, get dedicated personal time, and experience a sense of freedom from gendered interactions and dynamics (Berdychevsky et al., 2013). They experience a sense of community by meeting likeminded people and fostering friendships (Berdychevsky et al., 2016).

To complement these, travel companies include customizations such as accommodations with women-friendly floors or women-specific add-on services (Yang et al., 2018) to act as pull factors. Most of these services do not address the core issue of safety risks (Yang et al., 2018), and therefore are weak pull factors. Serious engagement with the issue is needed (Yang et al., 2018) to develop comprehensive solutions that would also act as strong pull factors for women travelers. Much research is required to identify and understand these pull factors.

This study identifies organizational purpose as a strong pull factor to motivate women to travel. It presents the case of Gotravel, a tourism organization in Bengaluru, India, which identified women safety as its purpose and aligned its internal norms and activities toward this purpose.

### About Gotravel

Established in 2013, and comprising of five women employees, including the founder and co-founder, Gotravel wanted to bridge the gap between women keen on traveling but concerned for their safety, and the untapped potential of India as a versatile tourist destination. Despite being a popular tourist destination, India does not attract the expected number of tourists (Khan et al., 2019), especially women. Consistent reports of India being unsafe for women (Charlton, 2014; World Travel and Tourism Council, 2014; Thomas and Mura, 2019), clubbed with underdeveloped infrastructure (Mohsin and Lockyer, 2010), has deterred women travelers from traveling to and in India. Reports of theft, sexual, and physical assault on tourists have further exacerbated negative perceptions of India (Agrawal, 2016; The Times of India, 2017).

Gotravel adopted women’s safety as its purpose to address these concerns. It aimed to encourage and equip women to travel through safe yet immersive travel experiences without restricting their destination choices. It provided women-only group tours and made extensive efforts to ensure safety. The founder and co-founder were directly involved in the vendor identification process. They visited destinations multiple times and personally verified safety-related arrangements at accommodations and with travel partners. They personally communicated with the families of their travelers to educate them about the safety measures. They conducted workshops with women on travel safety and travel-related skills such as riding bikes or fixing cars, which went beyond the immediate travel experience. Additionally, they also raised conversations on women safety in popular media and through traveler meet-ups, which included both men and women. Thus, they endeavored to create an ecosystem to reduce safety risks for women.

Internally, Gotravel consistently perpetuated its purpose through behavioral norms. These were also built into hiring, associating with external vendors, and work practices. Beginning with a few tours in the first year, Gotravel expanded to an average of 50–60 tours per year and has impacted over 7,000 lives. By aligning its norms and activities with its purpose, Gotravel was able to positively influence women’s perceptions of safety with respect to India, enhance their travel motivation, and develop long-term associations with them.

## Materials and Methods

Purpose was an emerging concept not examined in the context of the Indian tourism industry. In this context, an intensive study of a single unit would enable generation of new insights and enrich existing theory (Pettigrew, 1990; Yin, 1994; Lilius et al., 2011). Therefore, we employed the case study design (Yin, 1994) for our study. All of Gotravel’s activities, decisions, and stakeholder interactions were consciously aligned with its purpose, thus making it suitable for our study.

### Data Collection

Data were collected through a semi-structured interview protocol, site visits, and information on the organization’s website and social media platforms. The first author met the co-founder, visited their office, and also experienced their services as a customer. The first and second authors conducted semi-structured interviews with nine individuals associated with the organization. They interviewed the other co-founder, two employees handling communication and operations, and three vendors associated with Gotravel for at least 2 years, handling accommodation in South, East, and North India. They also connected with three customers associated with Gotravel for at least a year. Data from the organization’s website and social media platforms were used to corroborate interview data.

The interview protocol (Appendix A) was based on literature on positive organizations (Quinn, 2015) and purpose-driven organizations (Quinn and Thakor, 2018; Thakor and Quinn, 2019). Questions were classified into four groups: one focusing on general information about the participant and his/her association with the organization; second relating to the organization’s purpose; third examining organizational practices and norms that enforced the purpose; and fourth exploring participants’ loyalty and commitment to the organization. The questions were adjusted suitably for interviewing employees, customers, and vendors. Wherever needed, participants were probed further and were encouraged to provide specific examples to enhance the depth of the data. The interviews averaged 40 min and were recorded and transcribed verbatim.

### Data Analysis

Data were analyzed using thematic analysis for identifying patterns and developing themes (Braun and Clarke, 2006, 2012). The first two authors analyzed the data and then critically reviewed the identified themes with the third author. The first two authors read the interviews several times to familiarize themselves with the data. They worked independently to develop initial codes by identifying recurring patterns, similarities, and dissimilarities across interviews. Then, they critically reviewed the codes to explore commonalities and reconciled differences to develop a common set of initial codes. Next, they worked independently to group initial codes into themes by examining their relationships and inter-connections, and again engaged in mutual discussion to develop initial themes. They developed a set of themes, which they discussed with the third author for her inputs and identification of any individual biases. At this stage, they also examined their themes in light of extant literature on purpose. This enabled them to refine the themes and develop a comprehensive framework. This process of data collection and analysis incorporated data and researcher triangulation, thus ensuring the trustworthiness of emerging themes (Nowell et al., 2017). Table 1 presents the data analysis process and depicts changes in emergent themes during the different phases of data analysis.

**Table 1:** Data analysis process.

**Phase**	**Process description**	**Coders**
1. Data familiarization	Reading interviews multiple times	Authors 1 & 2
2. Initial coding	Independent coding to identify recurring patterns, similarities and differences in the data Inter-coder discussion to examine emerging patterns, and reconcile differences	Authors 1 & 2
3. Development of initial themes	Independent coding to group codes into themes, followed by inter-coder discussion to develop initial themes Initial themes and sub-themes: *Purpose:* Prosocial behaviour, meaningful experiences, beyond profit maximization *Communication:* Clarity, authenticity, transparency, honesty *Culture:* Empathy, trust, integrity *Practices:* Detail orientation, approachability, responsiveness	Authors 1 & 2
4. Theme consolidation and finalization	Discussion on themes in light of literature on purpose. Reviewing, refining and streamlining of themes Final themes and sub-themes: *Defining purpose:* Being Prosocial, creating a meaningful experience, going beyond profit maximization *Communicating purpose:* Clarity and authenticity, transparency and honesty *Embodying purpose*: Values: Empathy, trust, integrity, Practices: Being detail-oriented, approachability and responsiveness Themes brought together into an actionable framework	Authors 1 & 2, Reviewed with author 3

## Findings

**Figure 1:**
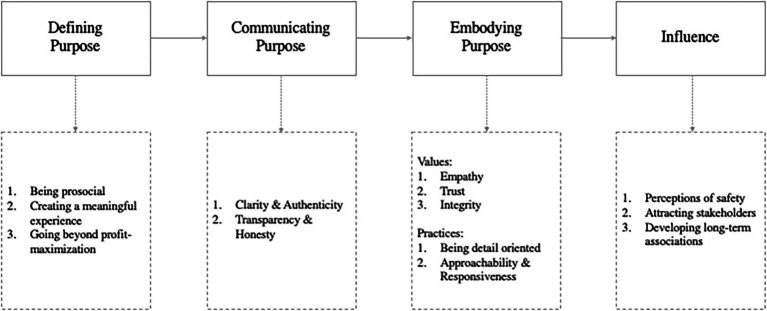
Implementing purpose in tourism organizations.

Analysis revealed three key elements of implementing purpose: defining purpose, communicating it, and embodying it. The resulting influence on internal and external stakeholders was identified. These elements were consolidated into a framework of implementing purpose and its outcomes (Figure 1), and are discussed below.

### Defining Purpose

Gotravel began with the idea of fulfilling a purpose of changing women’s travel experiences in India by facilitating safe, immersive tours. It used this purpose to extend conversations on women’s safety and continuously aligned and expanded its activities accordingly. Three key features assimilated in Gotravel’s purpose were being prosocial, creating a meaningful experience, and going beyond profit maximization. These were reflected in employee actions, service pricing, and customer experience.

#### Being Prosocial

Gotravel aimed to create social impact by helping and empowering women to travel with confidence. Thus, pro-sociality was inherent in its purpose. As a co-founder described as:

[We are] fulfilling a market need - providing tours and encouraging female travel. But we are also creating a social impact. For us, it’s one of the key driving factors.

Employees also described the organization as a closely associated community of people working towards helping women. An employee stated as:

I had the struggle of traveling solo, not being able to convince my parents, even lying to them to go to a different place. So I knew what women were going through, and now in the last three and a half years [of my employment], I have met people, I can sense that [Gotravel] is giving purpose to a lot of people who want to travel alone. There is this company or community that is helping them do it.

This was echoed by customers as well, who could identify the prosocial element as empowering women to travel, building their sense of confidence, and enabling them to feel free. A customer described as:

Their purpose is to get women out of their houses and travel and explore and not worry about safety and just give them some amount of freedom… that’s the reason why I was able to go with them.

#### Creating a Meaningful Experience

Gotravel aimed to create meaningful experiences for women travelers. In addition to carefully curated immersive travel plans, it also conducted trainings such as how to ride bikes or fix cars, which were useful life skills. According to the co-founder, such workshops aimed to create a meaningful impact that went beyond the short-term travel experience. This was corroborated by a customer:

… they do a lot more workshops like travel safety, biking, and fixing your car workshops, so it’s not just about traveling it’s also about how to live more confidently.

#### Going Beyond Profit Maximization

While Gotravel acknowledged that financial resources were necessary, its greater focus lays beyond profits on women travel. A co-founder emphasized the importance of the larger objective of the organization:

We actually started from social impact… of wanting to make a difference in terms of the way women travel in India. And from there, it grew into a business idea.

A similar understanding was seen among the employees who identified the key objective of the organization as not being profits:

It’s been six years now [Gotravel] is there, but when you are part of it, and you see the business side, I will not say they are very much money minded. Money is involved for our services [but] it never felt like we are doing it for the sake of money.

This was also built into the pricing of packages, which were comparatively lower than the competition while maintaining the quality of the tours. A customer appreciated this aspect:

It’s not a philanthropic organisation… if you compare the cost of the tour with any of the peers you would see that there is a distinct difference in the rate. It is a lot cheaper than any other tour and for the same quality.

### Communicating Purpose

Gotravel consistently reinforced its purpose both with internal and external stakeholders through communication. All communication was clear and authentic, and exhibited transparency and honesty. Communication was not always verbal in nature. It was also reflected in employee actions and behavior, indicating the internalization of purpose.

#### Clarity and Authenticity

Gotravel’s communications and actions were clear and authentic. Within the organization, purpose was not communicated explicitly. It was evident in the functioning of the organization and the clarity of functions to team members. As a co-founder mentioned as:

That’s [purpose] not definitely [stated] verbally, we do not do that… as a team, we are pretty sorted about our function…on what we want to do to help women travel.

Ultimately that’s the goal, and with each other’s action, our purpose gets revalidated.

That the organization was genuine in communicating its purpose was also evident in their efforts to reach larger audiences beyond immediate customers. A co-founder explained as:

We have our operations, marketing, and sales team who are working to reach mass audiences… colleges, schools, NGOs… to make them more confident and build trust in themselves that they can do anything that they want to do. So…it’s not only about working women who want to take off. It should be about the people who are in college and the short trips or workshops they can be a part of.

Clarity of purpose was also built into communications for external stakeholders through their website and social media. As a customer stated as:

Their communication is very good…I think they are pretty passionate about women’s travel and women’s empowerment and kind of enabling change in India’s travel space, I think she (a co-founder) does a really good job of communicating that through her personal timeline on Facebook as well as through the organization.

#### Transparency and Honesty

Communication across all organizational interactions were marked by transparency and honesty. This started with the hiring process during which the candidate was honestly informed about the organization. Often, it was the reason for individuals to join the organization. An employee described as:

When I first interacted with one of the co-founders, it was such an easy conversation because she was telling me all the pros and cons of [the organization], she was so transparent. It was the same with other members as well…. If a person is so transparent to you, you easily can connect to it (the purpose).

It created a sense of bonding that facilitated employees to learn quickly and start contributing to the organization. This also facilitated a working environment open to discussing issues and resolving them. As a co-founder explained as:

I think that honesty, about pointing out breakpoints it’s so apparent. There’s no fear of ruffling feathers, or there’s no fear of irritating someone. So you are generally very open about what’s working and what’s not working … that’s helping us move forward.

Transparency and honesty were also maintained in communication with customers. This, together with the purpose and experience overall, had such a positive impact on some customers that they offered their *pro-bono* services as tour guides. A customer stated as:

I think honesty is very important. Whatever they say is what they do, there is no hypocrisy. You know they are very transparent, so that is what I appreciate most about them [Gotravel] and also why I would love to continue being a trip lead.

### Embodying Purpose

Purpose was further integrated in Gotravel through practices and values. Several norms were developed to guide employee behavior and actions. Key values of empathy, trust, and integrity were identified. Practices of being detail-oriented, approachability, and responsiveness were observed.

#### Values

##### Empathy

Empathy enabled team members to understand where the other person was coming from. It created a supportive environment where conflicts and differences were handled smoothly. Members were encouraged to be who they were. Personal differences and goals were supported, which motivated them to work harder toward the organization’s goals and enhanced their dedication toward it. Such an environment led to employees feeling motivated and owning responsibility:

There is motivation from all of us to do different things, not just work-related… we are all connected to each other, which makes me part of this more than just for the company. So they kind of hold me accountable for a lot of things apart from work as well, and that makes it easier for me to wake up every day and do this work.

The organization also practiced empathy in their treatment of customers. They ensured positive experiences for customers, even when it costed them financially. Decisions were made keeping the mind the customer’s experiences and feelings. An employee illustrated as:

On one trip, the flights were delayed, flights are not our responsibility. When we announce a trip, we tell them what time to reach the trip’s starting location, reaching there is their job. But the flights got delayed, and the rest of the group had to move on, so the others had to have a separate vehicle organized for them to get dropped. Now it was not our fault, and it was not their fault either. But we thought it’s the start of the vacation, to make them pay [extra] money is going to sour the mood, and it just does not make anyone happy, so we pitched in half of it. We took a conscious hit, and it might sound silly and naive in the short-term, but in the long-term, we have ensured that they understand the kind of heart we put in the trip. It comes from the point of empathy, if we were in that position, our mood would be so off, we would be so upset that we might not be able to enjoy the rest of the trip.

##### Trust

The other core value of the organization was of building trust with all stakeholders. A co-founder stated that they trusted their employees from the time they joined, even though it impacted attrition rates such that only those who were comfortable with this amount of trust, stayed. They provided employees autonomy to work from any location, design, plan tours, and try new things at work. An employee remarked that this had a positive impact on her sense of ownership over her work. A tour lead also reflected the same sentiment:

When leading a trip, an amount of money is allotted, and as trip leads, we can do what we want. It’s just that we have to submit the bill, and the bill should not exceed what was quoted in the first place. And no question has ever been asked [about how and why the money was spent]. Whatever quote I have given has all been reimbursed; basically, they trust you.

Trust was also extended to customer interactions. A co-founder emphasized that they worked on building trust with their customers and did everything possible to never break it. The instance of the organization going the extra mile to accommodate travelers with a delayed flight and bearing half of the cost was a case in point.

The organization also focused on creating trust-based associations with their vendors. They actively engaged their vendors in tour planning and displayed trust through their financial dealing with them by settling payments in advance.

##### Integrity

Another crucial value displayed was integrity. It began with hiring, where prospective employees were clearly informed about the organization—one with limited financial resources but dedicated to enhancing women’s travel experiences. This attracted employees who were passionate about and motivated toward working for the organization’s purpose. As an employee stated as:

Whatever they say is what they do, there is no hypocrisy in what they do and what they say…that is what I appreciate most about the organization.

Similarly, customers’ expectations were realistically set before a tour commenced, so that they knew what they were getting into. A customer turned trip lead stated that the organization was honest and trustworthy, which was the reason for her volunteering her services to it.

#### Practices

##### Being Detail-Oriented

Stemming from their passion for travel and safety, Gotravel practiced detail orientation in all its activities. A co-founder mentioned that she loved planning trips and researching destinations, which was reflected in their itineraries that focused on off-beat destinations and travel paths. They also paid attention to customer demographics, interests, requirements, and expectations, building that into their itineraries. This was notably reflected in customers’ statements:

They ask you about the type of experience you are looking for, your age group, limitations, they go a little bit in-depth, which was nice, and after that, they came up with a detailed itinerary based on [these] inputs …the planning process was interactive and detailed.

The detailed level of planning for every tour was appreciated by vendors as well. A vendor described the interactive nature of planning and how it helped him in running his own business better:

Whatever ideas they incorporate, are passed onto me. The itineraries, the social media marketing… they share with me also. If an idea comes to them, they share with me, or if I come up with anything, [I share] …both ways we share the ideas, so it helps me also to grow. I am a male, in a package tour if a family comes, earlier I considered only from my limited perspective but after working with them I came to know what a lady accompanying on a trip looks [for] in a package, which I am learning from them.

##### Approachability and Responsiveness

Another important practice displayed was the approachability and responsiveness experienced by external stakeholders. This was reflected in the incident of a flight delay being quickly handled by the team, even when it was not part of their package. This was also evident in their interactive tour planning approach with both customers and vendors discussed above. All the customers interviewed were highly appreciative and mentioned instances of how the team, including the co-founder, was approachable and responsive to their requirements. They described as:

Customer 1: I really like the way they work, because the other tours I have gone with were not so friendly or approachable, but even the co-founder(s) have no airs about them. You can call her any time, and talk to her any time, she’s very approachable and then the feedback also they take up quite seriously.

Customer 2: I kept emailing them before I went on the trip, asking details about it, and they are very responsive. They keep you completely updated, and they put you on a WhatsApp group before you travel.

### Influence

Through effective definition, communication, and embodiment of purpose, Gotravel was able to positively influence its stakeholders. Perceptions of safety were enhanced, larger numbers of women were attracted to travel, thus facilitating their travel motivation, and positive long-term associations were developed.

#### Perceptions of Safety

By effectively implementing its purpose, Gotravel was able to enhance perceptions of safety among its customers. This was reflected in weekly testimonials from customers and media coverage, which the co-founder felt was validation for their efforts. This was also echoed by a customer:

…their purpose is to get women out of their houses and travel and explore and not worry about safety… The fact that it’s very easy for me to [travel with them], I do not even have to think about it I just have to say that I’m traveling.

#### Attracting Stakeholders

The implementation of the purpose was also reflected in the overall performance of the organization. In the first year of its operation, Gotravel conducted a few short tours. The company got positive feedback from the women who traveled with them, and through word of mouth, they were able to attract more customers. Over time, the company began organizing longer tours with customized itineraries, attracting a larger female customer base, including working mothers, teenagers, and ladies’ clubs. Thus, from a few short tours in the first year, the company expanded to conducting around 60 tours a year along with other social initiatives. Thus, through its purpose, Gotravel was able to enhance the travel motivation of customers and attract more women travelers.

#### Developing Long-Term Associations

Gotravel’s positive professional interactions with external stakeholders through communication, values, and practices led women travelers to appreciate the organization for what it stood for, and develop trust and comfort with them. It led them to travel again with the organization, some of them offering their services *pro-bono* as tour leads. Thus, it developed long-term associations with its customers.

## Discussion

The rise in women travelers globally (McNamara and Prideaux, 2010) has put focus on safety risks and constraints faced by them. In order to create an enabling travel environment and motivate women to travel, tourism organizations need to develop long lasting solutions to reduce safety risks (Yang et al., 2018). Extant research describes several push factors for women travel motivation but does not sufficiently discuss the pull factors. Pull factors become critical as tourism organizations can exercise greater control in planning and implementing them. This study contributes by presenting organizational purpose as a critical pull factor in motivating women to travel by addressing their safety issues. We identify key elements of defining and implementing purpose and consolidate it into a framework. This framework can act as a guide for tourism organizations looking to implement purpose effectively in order to create a safe travel environment for women and motivate them to travel.

Purpose is an organization’s reason for being (Collins and Porras, 1996) and directs all organizational actions. In the recent years, there is fresh emphasis on organizations to re-examine their purpose, go beyond economic value, and create societal value to build trust and lasting relationships with stakeholders (Hollensbe et al., 2014). Following this approach, Gotravel adopted a goal that went beyond profits to address a larger social concern of women travelers’ safety. Studies indicate that purpose can lead to various positive outcomes for the organization. It enhances employee perceptions of their behavior as being virtuous, aligned with important values, and provides meaning to their work (Rosso et al., 2010). This, in turn, can enhance their efforts (Birkinshaw et al., 2014; Quinn and Thakor, 2018), leading to higher job performance, organizational commitment (Liden et al., 2000), organizational identification, organizational citizenship behavior, and motivate them to work harder (Pratt and Ashforth, 2003; Michaelson et al., 2014). It can enable organizations to develop meaningful relationships with stakeholders (Gwartz and Spence, 2019) such as customers and enhance their loyalty and satisfaction (Du et al., 2013; Hainmueller and Hiscox, 2015).

Our findings corroborate these positive outcomes in Gotravel and the framework presents ways in which they can be achieved. Gotravel’s leaders had a strong sense of purpose, which was clearly defined and communicated both internally and externally. It was reinforced by values and practices employed, resulting in positive experiences for employees and customers. These experiences led to highly motivated employees and long-term relationships with customers. We further note that in order to reap benefits of purpose as a pull factor, it is important for organizations to not only define but also internalize it in practices and values. This happens when leaders believe in purpose, it is communicated authentically and employees put faith in it (Quinn and Thakor, 2018).

Our framework is useful for tourism organizations that have different identified purposes, such as eco-tourism, spiritual tourism, responsible tourism, or customer delight, and aim to operationalize it. It can assist them in aligning their activities with their goals, curate their business accordingly, and utilize purpose to enhance tourist motivation. In the context of the current pandemic, our study provides a way for tourism organizations to re-establish their trust with customers by reinventing themselves through purpose.

It should be noted that our framework is based on a single case and addresses a single type of purpose. While deploying it in other organizations, it is likely that variations in the identified features and additional unique features of implementing purpose may emerge due to differences in purposes and unique organizational characteristics. Therefore, while utilizing this framework, organizations should be mindful of such emergent variations and be open to incorporating them suitably.

Our study opens new research directions on purpose in the tourism sector. While we examined a single organization in depth, studying purpose in multiple organizations with larger number of stakeholders can deepen our understanding of the concept. Additionally, examining purpose as a variable quantitatively can further enrich literature.

## Data Availability Statement

The raw data supporting the conclusions of this article will be made available by the authors, without undue reservation.

## Ethics Statement

Ethical review and approval were not required for the study on human participants in accordance with the local legislation and institutional requirements. The patients/participants provided their written informed consent to participate in this study.

## Author Contributions

RC: conception of the study, research design, method, and interpretation. VL: literature review, data collection, and analysis. AT: introduction, method, interpretation, and discussion. All authors contributed to the article and approved the submitted version.

## Conflict of Interest

The authors declare that the research was conducted in the absence of any commercial or financial relationships that could be construed as a potential conflict of interest.

## Publisher’s Note

All claims expressed in this article are solely those of the authors and do not necessarily represent those of their affiliated organizations, or those of the publisher, the editors and the reviewers. Any product that may be evaluated in this article, or claim that may be made by its manufacturer, is not guaranteed or endorsed by the publisher.

## Appendix A

### Interview Protocol

#### General Information

1. What do you currently do?
2. What is your association with the organization?
3. For how long have you been associated with the organization?

#### Purpose

1. Does the organization have a higher purpose? How would you define it?
2. How do you identify with its purpose?
3. How do you see its purpose come to life in its functioning?

#### Factors That Reinforce and Facilitate Purpose

1. What enables the organization to carry out its purpose?
2. How is the organization’s purpose communicated? In terms of daily practices/functioning and more generally.
3. Are there any practices/activities carried out by the organization that enable it to fulfill its purpose? Can you elaborate on them?
4. How has this culture/environment been reinforced/cultivated since your association?

#### Influence on Stakeholders

1. Is there anything about the environment or culture of the organization that has encouraged you to continue your association with the organization?—As an employee/customer/vendor.
2. What attracted you to the organization?
3. What has made you decide to continue your association with the organization till now?
4. Is there anything else you would like to add?
